# Systematic Review and Meta-Analysis of the Use of Phosphodiesterase Type 5 Inhibitors for Treatment of Erectile Dysfunction following Bilateral Nerve-Sparing Radical Prostatectomy

**DOI:** 10.1371/journal.pone.0091327

**Published:** 2014-03-11

**Authors:** Xiao Wang, Xinghuan Wang, Tao Liu, Qianwen He, Yipeng Wang, Xinhua Zhang

**Affiliations:** 1 Department of Urology, Zhongnan Hospital of Wuhan University, Wuhan city, Hubei province, P. R. China; 2 Department of Cardiology, Zhongnan Hospital of Wuhan University, Wuhan city, Hubei province, P. R. China; 3 Department of Anesthesiology, Zhongnan Hospital of Wuhan University, Wuhan city, Hubei province, P. R. China; Eberhard-Karls University, Germany

## Abstract

Prostate cancer is relatively common cancer occurring in males. Radical prostatectomy (RP) is the most effective treatment for a localized tumor but erectile dysfunction (ED) is common complication, even when bilateral nerve-sparing RP (BNSRP) is performed. Clinical trials have shown varied effectiveness of phosphodiesterase type-5 inhibitors (PDE5-Is) for treatment of post-BNSRP ED, but there remains controversy over the application of this treatment and no formal systematic review and meta-analysis for the use of PDE5-Is for this condition has been conducted. This review was to systematically assess the efficacy and safety of oral PDE5-Is for post-BNSRP ED. A database search was conducted to identify randomized controlled trials (RCTs). The comparative efficacy of treatments was analyzed by fixed or random effect modeling. Erectile function was measured using the International Index of Erectile Function (IIEF), Sexual Encounter Profile (SEP) question-2, 3 and the Global Assessment Question (GAQ). The rate and incidence of adverse events (AEs) were determined. The quality of included studies was appraised using the Cochrane Collaboration bias appraisal tool. Eight RCTs were included in the analyses. PDE5-Is were effective for treating post-BNSRP ED compared to placebo when erectile function was determined using the IIEF score [mean difference (MD) 5.63, 95% confidence interval (CI) (4.26–6.99)], SEP-2 [relative risk (RR) 1.63, 95% CI (1.18–2.25) ], SEP-3 [RR 2.00, 95% CI (1.27–3.15) ] and GAQ [RR 3.35, 95% CI (2.68–4.67) ]. The subgroup analysis could find a trend that longer treatment duration, higher dosage, on-demand dosing, sildenafil and mild ED are associated with more responsiveness to PDE5-Is. PDE5-Is were overall well tolerated with headache being the most commonly reported AE. Our data provides compelling evidence for the use of PDE5-Is as a primary treatment for post-BNSRP ED. However, further studies are required to optomize usage parameters (such as dosage and duration of treatment).

## Introduction

Prostate cancer is a relatively prevalent disease, and in some Western countries it is the leading type of malignant tumor diagnosed in males [1]. However the prognosis is good, with a 5-year relative disease-specific survival rate of approximately 100% for patients who undergo localized cancer treatment by radical prostatectomy (RP) [2]. The number of RPs has been increasing annually, with the average age of treated patients decreasing [3]. Erectile dysfunction (ED), is the most common complication in patients undergoing RP, which can have a significant negative impact on patients' health-related quality of life and wellbeing [4]. Even when bilateral nerve-sparing RP (BNSRP) procedures are performed around 15%–80% of men experience postoperative ED [5], [6]. More patients would accept this surgical treatment if it were not for the possibility that they will develop ED postoperatively [7]. Many factors influence the incidence and severity of postoperative ED, including patient age, tumor stage, preoperative potency, length of time following surgery and the experience of surgeon [8]–[12]. The pathophysiology of post-RP ED mainly results from three causes; neural injury, vascular injury, and smooth muscle damage [13], [14]. Thermal injury to the cavernous nerves will result in permanent loss of potency after surgery and traction on the nerves may also be just as deleterious. Vascular injury primarily involves damage to the accessory pudendal arteries. It has also been well documented in several studies that smooth muscle and endothelium undergo structural changes resulted from neurapraxia [15]. Of note, smooth muscle apoptosis and upregulation of collagen expression are the primary conditions resulting in venous leak [16]–[18].

Post-RP ED may take up to 4 years to resolve, with as many as 20–80% of these patients never returning to normal erectile function [19]. The incidence of complete ED has been reported to be 26–100% and partial ED 16–48% [20]. The aforementioned new insights into the pathophysiology of post-RP ED have led to the development of penile rehabilitation strategies, which is defined as the use of any drug or device at or after RP to maximize erectile function recovery. These strategies include phosphodiesterase type 5 inhibitors (PDE5-Is), intracavernosal injections, intraurethral alprostadil, vacuum constriction devices (VCD), neuromodulatory therapy or a combination of these treatments [21], [22].

The advent of PDE5-Is has revolutionized ED treatment with an average success rate of 60–70% in the general patient population [19], [23]. PDE5-Is are more commonly used in rehabilitation programs than other treatment options, and are often the first line of treatment [15], [24]. The efficacy and side effects of PDE5-Is used to treat ED subsequent to BNSRP have been extensively studied. However, varying efficacy has been reported with no definitive evidence to support the optimal treatment strategy, such as dosage, onset and duration of use, as well as efficacy of the different PDE5-Is. Moreover, at present there is no consensus or guidelines on their use and no formal systematic review and meta-analysis have been conducted. In this review, we apply the methods of evidence-based medicine to evaluate and analyze the documented trials of PDE5-Is to treat post-BNSRP ED so as to provide a more systematic and comprehensive assessment of their use and efficacy.

## Materials and Methods

### Inclusion criteria

#### Trial design

Randomized controlled trials (RCTs) were included and analyzed accordingly.

#### Type of participants

Patients who underwent BNSRP or were scheduled to undergo BNSRP, who were in stable heterosexual relationships, had no residual tumor, no history of cardiovascular disease, no prejudice for the use of PDE5-Is, no postoperative chemo- or radio-therapy, and who were treated with PDE5-Is for ED in a prospective trial design.

#### Type of interventions

PDE5-Is (sildenafil, vardenafil, tadalafil, avanafil, lodenafil, mirodenafil, udenafil)or placebo were orally administered using any regimen and for any duration.

#### Type of outcome measures

Erectile function was measured with International Index of Erectile Function (IIEF), Sexual Encounter Profile question 2 (SEP-2, “Were you able to insert your penis into your partner's vagina?”[yes/no]), Sexual Encounter Profile question 3 (SEP-3, “Did your erection last long enough for you to have successful intercourse?”[yes/no]) and the Global Assessment Question (GAQ, “Has the treatment you have been taking during this study improved your erection?”[yes/no]).

#### Adverse Events (AEs)

The rate of AEs was determined.

All the aforementioned inclusion criteria are necessary for selection of studies

### Exclusion criteria

Repeat publications, sample size<10 and where studies were only reported superficially, such as in the form of an abstract.

### Data sources and searches

We performed database searches of Cochrane Library (Issue 6, June 2013), PubMed (1966-June 2013), Embase (1984-June 2013), AMED (1985-June 2013), CINAHL (1966-June 2013) and the National Health Service Research Register (1990-June 2013) using the following keywords in combination with both medical subject headings terms and text words: phosphodiesterase type 5 inhibitor *or* tadalafil *or* sildenafil *or* vardenafil *or* avanafil *or* lodenafil *or* mirodenafil *or* udenafil *plus* erectile dysfunction *plus* radical prostatectomy. There was no limitation on publication status or language. Reference lists of the included studies were checked manually to further identify related studies.

### Selection of studies

Three reviewers (TL, QWH and YPW) independently screened the title, abstract and keywords of each article retrieved. Full-text papers were screened for further assessment if the information given suggested that the study fulfilled the inclusion criteria and did not meet the exclusion criteria. Where differences in opinion existed, they were resolved through open discussion.

### Bias assessment

The methodological quality of included studies was appraised with the Cochrane Collaboration bias appraisal tool. In particular, the following factors were evaluated: (1) Adequate sequence generation? (2) Allocation concealment? (3) Binding? (4) Incomplete outcome data addressed? (5) Free of selective reporting? (6) Free of other bias?

Every question was answered with “yes”, “no” or “unclear” and three reviewers (TL, QWH and YPW) assessed each trial. In case of disagreement, judgment was made through open discussion.

### Assessment of evidence quality

The quality of evidence on IIEF score, SEP2, SEP3, GAQ and AEs were evaluated using the grading of recommendation assessment development and evaluation (GRADE) system. The quality of evidence was presented as follows: (1) high, indicating further research is very unlikely to change our confidence in the estimated effect; (2) moderate, indicating further research is likely to have an important impact on our confidence in the estimated effect and may change the estimate; (3) low, indicating further research is very likely to have an important impact on our confidence in the estimated effect and is likely to change the estimate; (4) very low, indicating that the estimate is very uncertain.

Even though evidence based on RCTs is regarded as high quality, it can be reduced with the following factors: (1) limitation of study design; (2) unaccountable heterogeneity; (3) indirect evidence; (4) inaccurate outcomes; (5) reporting biases.

### Data extraction

Data were extracted independently by three reviewers (TL, QWH and YPW) using a standard form, including study characteristics (title, publication time, and sample size), patient characteristics (age, height, weight, race), intervention, control, method (randomization, blinding, and loss to follow up), and outcomes (estimates, standard error, and *p* value). Discrepancies were resolved by discussion. The authors of original studies were consulted for missing information where necessary.

### Data synthesis and analysis

The comparative effects were analyzed by meta-analysis method using Cochrane Collaboration review manager software. Heterogeneity among studies was assessed with the Chi^2^ test and the I^2^ index statistic. If p>0.1 and I^2^<50%, which meant homogeneity existed among studies, we would use fixed-effect models for the calculation of pooled effect index and if p<0.1 and I^2^>50%, random-effect models would be applied. Subgroup analysis was performed by stratifying the treatment duration, drug delivery, dosage and type of PDE5-Is. In the subgroup analysis, we pooled the effect of these subgroups separately, but in the analysis of the overall effect of the PDE5-Is group, we incorporated the data of different subgroups into one verum arm. We used F test for multigroup comparisons and T test for the pair group comparison.

## Results

### Characteristics of included studies

**Figure 1:**
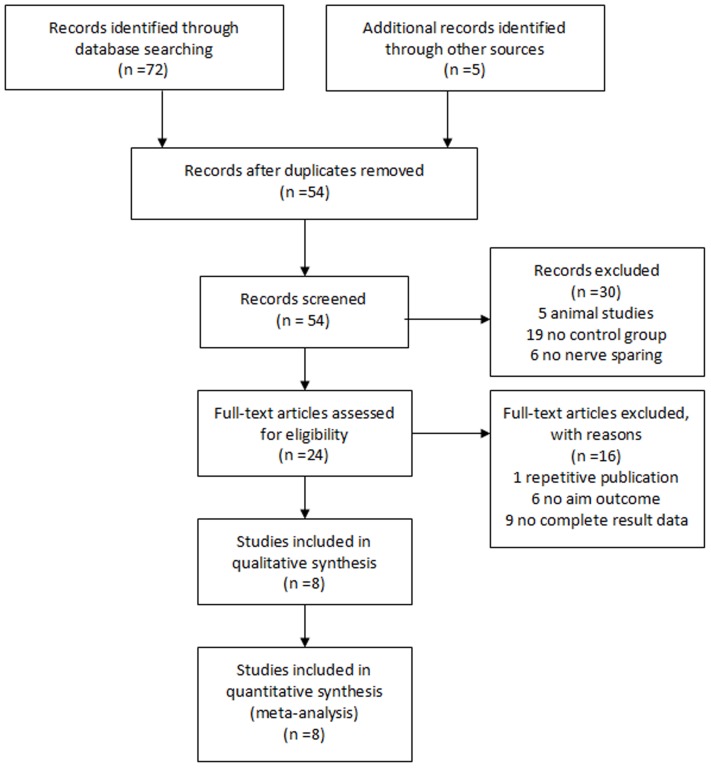
Flowchart of identification and selection of studies for the systematic review.

Using the database search strategy, a total of 77 records were retrieved from Cochrane Library, PubMed, Embase, AMED, CINAHL and the National Health Service Research Register, of which eight RCTs [25]–[32] finally met full inclusion criteria for this review. There were no original clinical trials studied the new generation of PDE5-Is (lodenafil or mirodenafil or udenafil) for post-RP ED. Fig. 1 shows the search process. Table 1 provides details of the included trials.

**Table 1:** Characteristics of the included studies in meta-analysis.

Study ID	Country	Sample size	Inclusion criteria	Intervention	Control	Treatment period	Outcome	Adverse event
Montorsi et al 2008	Multicentre across Europe, USA, Canada and South Africa	628	Patients scheduling to undergo BNSRRP	(i) vardenafil 10 mg nightly plus on-demand placebo (ii) flexible-dose vardenafil on-demand plus nightly placebo	nightly placebo plus on-demand placebo	9 months	rate of IIEF score ?22 and SEP-3 success rate	Included
Aydogdu et al 2011	Turkey	85	Patients scheduling to undergo BNSRRP	tadalafil 20 mg/day	no use of tadalafil	6 months	IIEF score, SEP-2 success rate, SEP-3 success rate and penile measurements	N/A
Padma-Nathan et al 2008	Multicentre across North America and France	125	Patients scheduling to undergo BNSRRP	sildenafil 50 mg or 100 mg nightly	placebo nightly	9 months	rate of responders, IIEF score and the duration of penile tumescence and rigidity	Included
Brock et al 2003	Multicentre across USA and Canada	440	Patients with ED 6 months to 5 years after NSRRP	vardenafil 10 mg or 20 mg on-demand	placebo on-demand	3 months	IIEF score, SEP-2 success rate, SEP-3 success rate and GAQ success rate	Included
Montorsi et al 2004	Multicentre across Canada, Germany, Italy, Netherlands, Spain, USA and UK	303	Patients with ED 12 months to 48 months after NSRRP	tadalafil 20 mg on-demand	placebo on-demand	3 months	IIEF score, SEP-2 success rate, SEP-3 success rate, GAQ success rate and EDITS score	Included
Mulhall et al 2012	USA	298	Patients with ED 6 months or more after NSRRP	avanafil 10 mg or 20 mg on-demand	placebo on-demand	3 months	SEP-3 success rate, GAQ success rate	Included
Bannowsky et al 2008	USA	43	Patients scheduling to undergo BNSRRP	sildenafil 25 mg nightly	no use of PDE-5 inhibitors	13 months	IIEF score and SEP-3 success rate	N/A
Cavallini et al 2005	Italy	96	Patients with ED 6 months or more after NSRRP	(i) ALC+PLC+sildenafil 100 mg on demand (ii) sildenafil 100 mg on-demand	placebo on-demand	4 months	IIEF score and parameters of cavernosal arteries	Included

### Risk of bias

As described in Table 2, four of the eight included studies [25], [27]–[29] had adequate randomization according to the Cochrane Collaboration bias appraisal tool. One study [31] was randomized according to preoperative IIEF score, age and number of nocturnal erections and status of BNSRP. The other three did not describe their randomization method. Only one study [32] showed method of allocation concealment and the others did not describe their approach. Both patients and researchers were reported as blinded in six studies [25], [27]–[30], [32], but did not report the blinding method. Three studies [25], [26], [31] did not provided complete outcome data and one study [25] did not report all design outcomes. All eight studies were free of other bias.

**Table 2:** Risk of bias summary.

Study ID	Adequate sequence generation?	Allocation concealment?	Blinding?	Incomplete outcome data addressed?	Free of selective reporting?	Free of other bias?
Montorsi et al 2008	Y	U	Y	N	N	Y
Aydogdu et al 2011	U	U	U	N	Y	Y
Padma-Nathan et al 2008	Y	U	Y	Y	Y	Y
Brock et al 2003	Y	U	Y	Y	Y	Y
Montorsi et al 2004	Y	U	Y	Y	Y	Y
Mulhall et al 2012	U	U	Y	Y	Y	Y
Bannowsky et al 2008	N	U	U	N	Y	Y
Cavallini et al 2005	U	Y	Y	Y	Y	Y

Review authors' judgments about each risk of bias item for included study. Y =  yes; N =  no; U  =  unclear.

### Evidence quality

The present systematic review shows five meta-analysis outcomes including scores of IIEF, SEP-2 success rate, SEP-3 success rate, GAQ success rate and AEs incidence, of which the IIEF score, SEP-2 success rate and SEP-3 success rate were of moderate quality evidence, while the others were high quality evidence, as evaluated by the GRADE system.

### Efficacy of PDE5-Is

**Figure 2:**
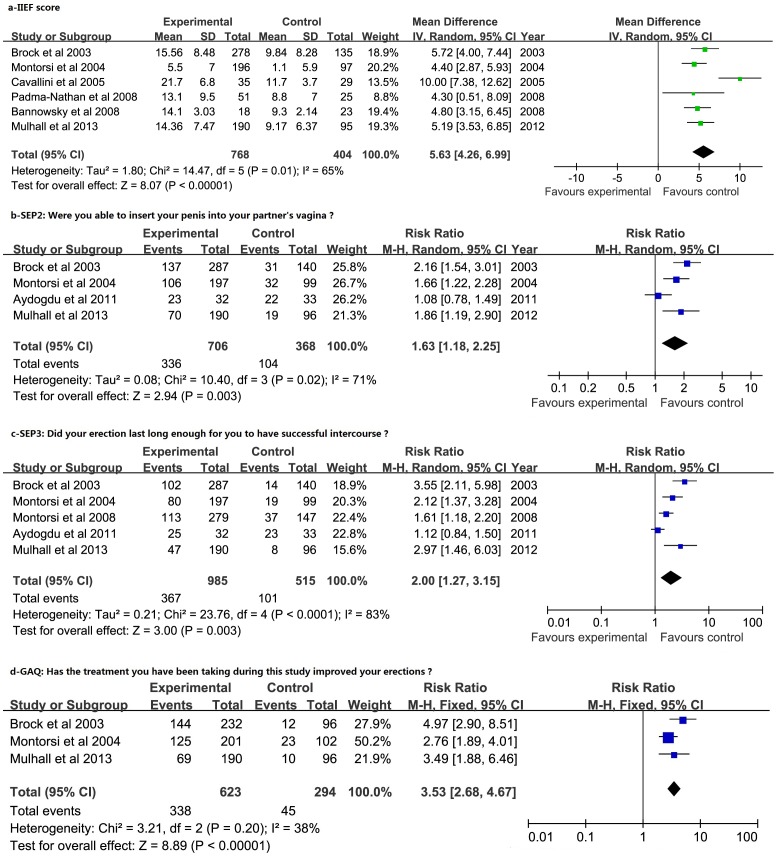
Forest plot for meta-analysis of efficacy of PDE5-Is versus placebo by assessment of IIEF, SEP-2, SEP-3 and GAQ. a: Mean difference (MD) of score of the International Index of Erectile Function (IIEF); b: Relative risk (RR) of success rate by Sexual Encounter Profile question 2 (SEP-2, “Were you able to insert your penis into your partner's vagina?”[yes/no]); c: Relative risk (RR) of success rate by Sexual Encounter Profile question 3 (SEP-3, “Did your erection last long enough for you to have successful intercourse?”[yes/no]); d: Relative risk (RR) of success rate by Global Assessment Question (GAQ), “Has the treatment you have been taking during this study improved your erection?”[yes/no]) with 95% confidence interval (CI) for the studies on phosphodiesterase type-5 inhibitors (PDE5-Is) versus control arms

The I^2^ standing for the heterogeneity among the studies was 65%, 71%, 83% and 38% for IIEF score, SEP-2, SEP-3 and GAQ, respectively. A random-effect model was used for the first three measurements while a fix-effect model was applied to GAQ. As shown in Fig. 2a, six studies [27]–[32] included scores of IIEF. The mean difference (MD) for IIEF was 5.63 [95% confidence interval (CI) 4.26 to 6.99] in favor of the PDE5-Is arm. Sensitivity analysis was performed by excluding each of the six studies, respectively, and the pooled effect data had statistical significance. Fig. 2b provides details of four studies [25], [26], [28], [30] including the number of participants answering “yes” to the question “Were you able to insert your penis into your partner's vagina?” The relative risk (RR) for answering “yes” was 1.63 (95% CI 1.18 to 2.25) in the PDE5-Is group when compared to controls. In the sensitivity analysis, the study of Montorsi 2004 [29] was excluded and the pooled RR was 1.62 (95% CI 0.99 to 2.64), which indicates no statistical significance. Fig. 2c provides details of five studies [25], [26], [28]–[30] including the number of participants answering “yes” to the question “Did your erection last long enough for you to have successful intercourse?” The RR for answering “yes” was 2.00 (95% CI 1.27 to 3.15) in the PDE5-Is group when compared to controls. Sensitivity analysis was performed by excluding each of the five studies and the pooled effect data had statistical significance. Fig. 2d provides details of three studies [28]–[30] included the number of participants answering “yes” to the question “Has the treatment you have been taking during this study improved your erection?” The RR for answering “yes” was 3.53 (95% CI 2.68 to 4.67) in the PDE5-Is arm when compared to placebo. Sensitivity analysis was performed by excluding each of the three studies and the pooled effect data had statistical significance.

### Subgroup analysis

**Figure 3:**
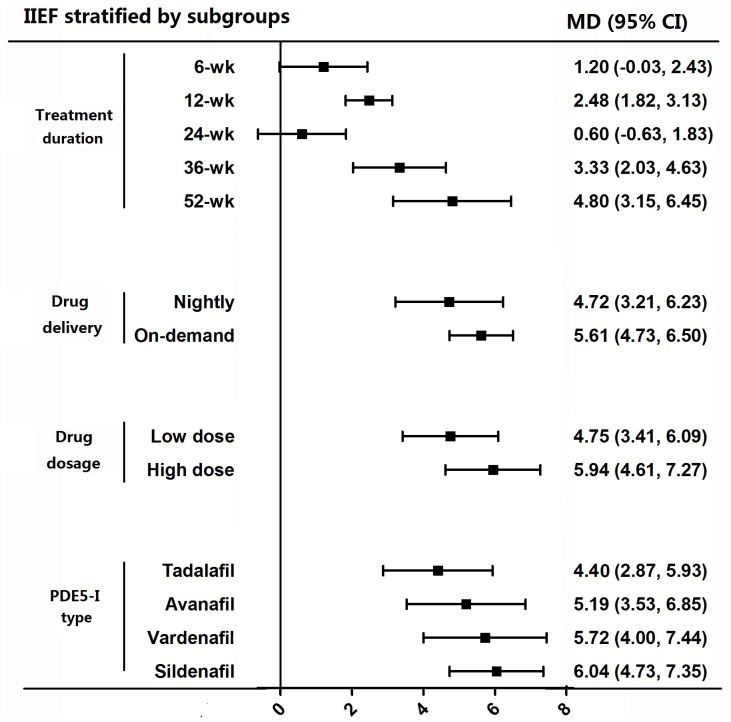
Mean difference (MD) of score of the International Index of Erectile Function (IIEF) stratified by different subgroups. Treatment course was stratified to 6, 12, 24, 36 and 52 weeks. Drug delivery was stratified to nightly and on-dmand dosing. Drug dosage was stratified to low dose and high dose subgroups. PDE5-Is type was stratified to tadalafil, avanafil, vardenafil and sildenafil.

Bannowsky et al [31] followed-up patients at 6, 12, 24, 36 and 52 weeks post surgery and found that the significant difference between the sildenafil (25 mg) arm and controls began at 36 week and the IIEF score continued to increase at 52 week. Brock et al [28] and Montorsi et al [29] set a 12-week treatment period, while Padma-Nathan et al assessed the participants at 36 weeks. We pooled the MD of IIEF for various treatment duration and showed a trend of more responsiveness to PDE5-I with longer treatment duration, but no statistical significance could be obtained (F = 1.28, p = 0.28). The MD of IIEF was 1.2 (95% CI −0.03 to 2.43), 2.48 (95% CI 1.82 to 3.13), 0.6 (95% CI −0.63 to 1.83), 3.33 (95% CI 2.03 to 4.63) and 4.8 (95% CI 3.15 to 6.45) at 6, 12, 24, 36 and 52 weeks, respectively (Fig. 3). Montorsi et al compared the efficacy of vardenafil used nightly with on-demand dosing and the proportion of patients with an IIEF score ≥22 was greater in on-demand group than in nightly dosing group (p = 0.0065), suggesting vardenafil had better effect when on-demand administered. We pooled the results of six studies according to drug delivery. The MD of IIEF was 5.61 (95% CI 4.73 to 6.50) for on-demand dosing and 4.72 (95% CI 3.21 to 6.23) for administered nightly (Fig. 3), but no statistical significance could be obtained (t = 0.64, p = 0.52). Three studies [27], [28], [30] had dosage subgroups (vardenafil 10 mg vs 20 mg, sildenafil 50 mg vs 100 mg and avanafil 100 mg vs 200 mg) and these studies showed a trend that higher dose group seemed to be more effective, but no statistical significance could be obtained (t = 1.24, p = 0.22). The pooled MD of IIEF was 4.75 (95% CI 3.41 to 6.09) for lower dose and 5.94 (95% CI 4.61 to 7.27) for higher dose (Fig. 3). We also compared the efficacy of different types of PDE5-Is and found a trend that sildenafil had a tendency to appear more efficacious than others with a pooled MD of IIEF of 6.04 (95% CI 4.73 to 7.35), followed by vardenafil, avanafil and tadalafil with a MD of 5.72 (95% CI 4.00 to 7.44), 5.19 (95% CI 3.53 to 6.85) and 4.4 (95% CI 2.87 to 5.93), respectively (Fig. 3), but on significance could be obtained, either (F = 0.51, p = 0.68). Mulhall et al [30] stratified patients into three subgroups based on them having mild, moderate or severe ED. This study showed that the improvement of ED by PDE5-Is negatively related to the severity of ED (IIEF score increased by 5, 4.7 and 4.5 for mild, moderate and severe ED groups, respectively). Brock et al [28] applied the same stratification and consistently found that the increase of IIEF score was from 20 to 25.5,13 to 20.8 and 7 to 11.9 for mild, moderate and severe ED groups, respectively. Montorsi et al [29] defined a subgroup of patients as presenting evidence of postoperative penile tumescence and found that this subgroup had an increase in IIEF score of 5.9±0.7, while patients without postoperative penile tumescence had an increase in IIEF score of 4.1±0.9. As the lack of original data, we cannot pool the results by stratifying with the severity of ED. There is evidence from one trial only [32] that PDE5-Is are more effective when combined with NO donors, acetyl-L-carnitine (ALC) and propionyl-L-carnitine (PLC).

### Adverse events

**Figure 4:**
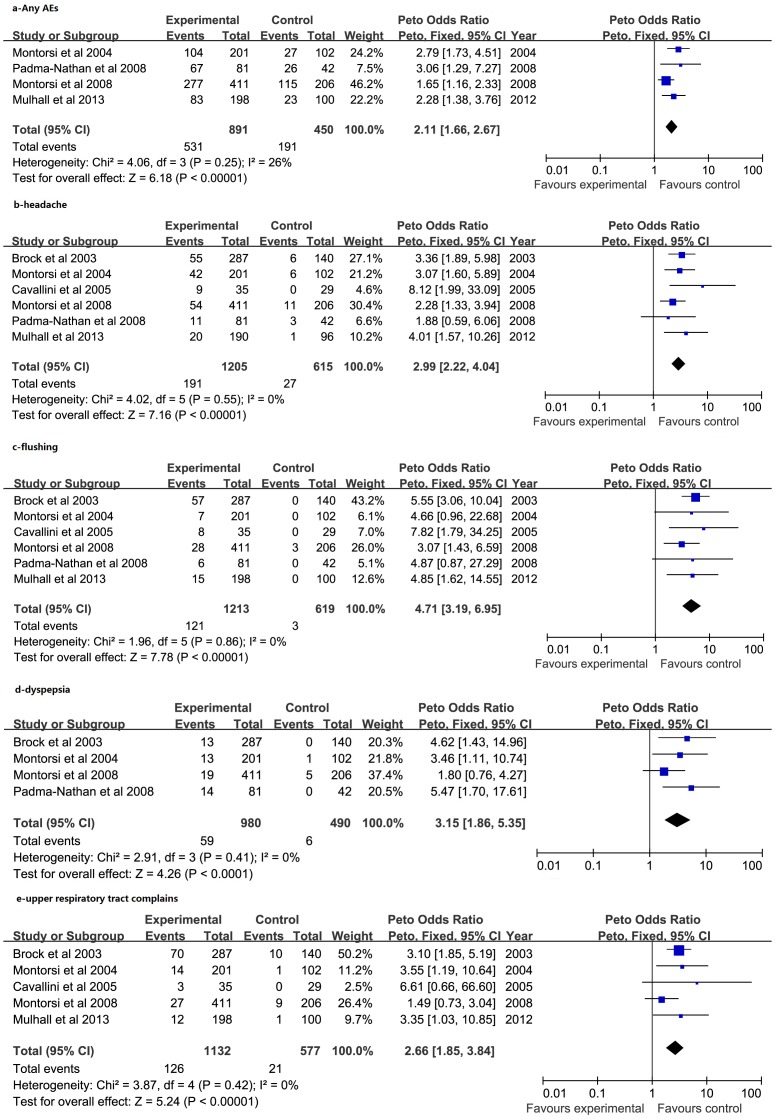
Forest plot for meta-analysis of adverse events of PDE5-Is. Peto odds ratio (OR) of incidence of any adverse events (a); headache (b); flushing (c); dyspepsia (d) and upper respiratory tract complains (e).

Six studies [25], [27]–[30], [32] reported the number of AEs, of which four studies [25], [27], [29], [30] reported all AEs. Fig. 4a shows a total 531 of 891 patients suffering AE in the PDE5-Is arm compared to 191 of 450 in controls, a risk ratio of 2.11 (95% CI 1.66 to 2.67). Among the AEs, headache (Fig. 4b) was the most frequent event reported, with a total of 191 of 1205 patients in the PDE5-Is arm compared to 27 of 615 in controls with a risk ratio of 2.99 (95% CI 2.22 to 4.04). Other common AEs were flushing, dyspepsia and upper respiratory tract complains (Fig. 4c,d,e), with an odds ratio of 4.71 (95% CI 3.19 to 6.95), 3.15 (95%CI 1.86 to 5.35) and 2.66 (95% CI 1.85 to 3.84), respectively.

## Discussion

This is the first meta-analysis and systematic review on the use of oral PDE5-Is for treating ED subsequent to BNSRP. Overall our results demonstrate that PDE5-Is were efficacious and well tolerated by this specific ED patient population. Headache was the most frequently reported adverse effect. Our review also showed trends that higher dose, longer duration of treatment, on-demand dosing, sildenafil and mild ED are associated with a greater efficacy of PDE5-Is to treat ED.

The overall quality of the included studies is acceptable. Only one study was randomized according to preoperative IIEF score, age and number of nocturnal erections and status of BNSRP and judgment “no” for the assessment of adequate sequence generation was given to this trial. Most studies did not describe their allocation method in details and this may be a risk factor for the methodological quality. Also most studies did not report their blinding approach. The study of Montorsi [25] did not report had a higher withdrawal rate, And the studies of Aydogdu [26] and Bannowsky [31] followed-up did not provide adequate reasons for attrition, and judgment “no” was given for the assessment of incomplete data addressed. All eight included studies had no severe imbalanced baseline, early withdrawal, or other recognizable risk of bias. Thus positive judgment was made for the assessment of free of other risks.

The trial design scheme of the included studies could be divided into two types. The first design had trial participants scheduled to undergo BNSRP and then treated shortly after surgery (after removal of catheter or one month post-RP). The second design had participants who had already undergone BNSRP and subsequently complained of postoperative ED before undergoing therapy. Both trial design schemes had flaws. In the first study design not all the patients would experience post-RP ED. Thus the assessment of the efficacy of PDE5-Is for ED potentially had some bias due to inclusion of some patients without ED. In the second type of trial, patients had already undergone prostatectomy, and whilst all of them had ED, some of them could have been previously treated for ED prior to enrollment in the trial. The authors enrolled both previously treated and untreated patients in the study to enlarge sample size, however, previous treatments could have potentially influenced the efficacy of the subsequent intervention. In terms of the trial design, five of the included studies [25]–[27], [31], [32] were of the first design scheme and three studies [28]–[30] belonged to the second. In fact, we have done the subgroup analysis stratified with different design schemes, but no significant difference were found (data not shown). Although our review showed PDE5-Is were effective for both type of trial design schemes (treatment almost immediately after RP or later), early treatment is suggested [33], [34].

The different trial design schemes may be a source of the heterogeneity in the analysis of IIEF score, SEP-2 and SEP-3 success rate. However other outcomes measures demonstrated good homogeneity, such as the rates of adverse events. This may be due to the determination of IIEF score, SEP-2 success rate and SEP-3 success rate being subjective parameters, leading to inconsistency in the outcome assessment. Although these subjective parameters were determined with validated questionnaires, there were clinical or methodological diversity among studies resulting in heterogeneity. Even though some included studies [26], [32] assessed objective outcomes, such as the hemodynamics index, it was not possible to pool the outcomes by meta-analysis due to the limited number of studies.

Of the meta-analysis outcomes, only the SEP-2 was unstable. As the study of Montorsi 2004[29] had a large weight (26.7%) and it had some unclear bias, the pooled RR was 1.62 (95% CI 0.99 to 2.64) after excluding this study, indicating no statistical significance. Thus the conclusion about the intervention effect on SEP-2 should be considered with caution. The other meta-analysis outcomes were stable.

Our review clearly demonstrates that treatment with PDE5-Is gave statistically significant clinically favorable outcomes in term of IIEF scores, SEP-2, SEP-3 and GAQ success rate when compared with placebo. Similarly, the meta-analysis from Candy B, et al [35] showed oral PDE5-Is were effective in the medium term (up to 4 months) when used to treat ED subsequent to external beam radiotherapy or radical bilateral nerve-sparing or unilateral nerve sparing RP for prostate cancer. However, no significant differences were found in their comparisons of the PDE5-Is dose, or between patients with unilateral or bilateral nerve sparing prostatectomy. They attributed these observations to too few patients in each subgroup. In our subgroup comparisons, there was a trend that higher dose, longer course of treatment, on-demand dosing and sildenafil were associated with more efficacy of PDE5-Is, but these trends were not sufficient to demonstrate statistical differences. The lack of statistical significance could also be accounted for by insufficient patient numbers in the trials included in the present review. In addition, we incorporated the data of different subgroups into one verum arm in the analysis of the overall effect of the PDE5-Is, the different interventions may be also a source of heterogeneity. As the vast majority of patients after BNSRP experience severe ED, the efficacy of PDE5-Is in this population would not be expected to be high as in the general population of ED patients. In Brock's study [28] only 28% severe patients had successful intercourse at the end of treatment. In addition, in the studies used for this analysis no direct comparisons were performed between the various PDE5-Is. Instead, we made indirect comparison with different type of PDE5-Is in the subgroup analysis and found sildenafil seemed more effectiveness than the others, but no statistical difference either. It is also interesting to note that on-demand dosing may be more effective in improving IIEF score than when administered nightly. Furthermore, the subgroup analysis indicated that patients with severe ED were less sensitive to PDE5-Is. Similarly, in Brock's study [28], PDE5-Is were found less effective in patients who underwent unilateral nerve-sparing RP compared to BNSRP. Since PDE5-Is improve erectile function depending on the peripheral release of nitric oxide from cavernosal nerve terminals, the neural defect could weaken the efficacy of PDE5-Is. Therefore, neural factor maybe play a more important role in the development and progression of ED after RP, rather than vascular factor or other factors.

Since PDE5-Is ability to stimulate an erection require the presence of nitric oxide (NO) in the penis, one study investigated if it would be beneficial to combine PDE5-Is with NO donors such as ALC and PLC, and demonstrated improved efficacy with this strategy [32]. Overall, the results of our review provide evidence for the efficacy of PDE5-Is as a primary rehabilitation treatment for ED following BNSRP.

Most studies raised concern over cardiovascular safety [36]–[38] even though some studies reported that PDE5-Is may have a beneficial effect on cardiovascular system [39], [40]. In our systematic review, no trial reported severe cardiovascular AEs. The total incidence of AEs associated with PDE5-Is was greater than placebo. However, in most cases treatment-related AEs were mild to moderate in nature, and the overall safety profile of these drugs was good. When prescribing PDE5-Is, physicians should make patients aware of their common complications, such as headache, flushing, dyspepsia and upper respiratory tract complaints.

## Conclusion

PDE5-Is were determined as efficacious and well tolerated for treatment of ED subsequent to BNSRP and early initiation of treatment is recommended. Also our subgroup analysis showed a trend that higher dose, longer course of treatment, on-demand dosing and mild ED are associated with greater responsiveness to PDE5-Is. Additionally, direct comparisons among various PDE5-Is were not available and indirect comparison made in current review found a trend that sildenafil was more effectiveness than the others. Statistical significance for these trends could not be obtained in the subgroup analysis, probably due to insufficient patient numbers. Therefore, to provide sound practical advice for the use of of PDE5-Is for post-BNSRP ED, such as when to initiate treatment, what dosage to use, duration of treatment, selection criteria and which drug is most efficacious, more clinical trials are required. A high degree of heterogeneity was observed in the studies analyzed. Therefore, we recommend close attention to trial design and determination of more objective outcome measurements in future studies.

## Supporting Information

Checklist S1 **PRISMA 2009 Checklist.**
(DOC)
